# Identification of Molecular Substrate for the Attenuation of Anxiety: A Step Toward the Development of Better Anti-Anxiety Drugs

**DOI:** 10.1100/tsw.2001.33

**Published:** 2001-05-01

**Authors:** Uwe Rudolph

**Affiliations:** Institute of Pharmacology and Toxicology, University of Zürich., Switzerland

**Keywords:** benzodiazepines, diazepam, GABA recepator, ion channel, mutagenesis, knock-in-mice, anxiety, anxiolysis

## Abstract

Anxiety disorders affect some 19 million people in the U.S. alone, costing $46.6 billion, or one third of the nation’s total mental health bill in 1990. Benzodiazepine tranquilizers like the prototypic diazepam are among the most widely used anti-anxiety agents. In addition to their anxiolytic action, they also induce sedation and may impair motor coordination, both of which are undesired side effects when they are used as anxiolytics. Not surprisingly, road traffic accidents may be increased for patients on classical benzodiazepines. In addition, these drugs carry the risk of dependence liability. Benzodiazepines augment the action of the inhibitory neurotransmitter g-aminobutyric acid (GABA) at contact points between two nerve cells called synapses, points at which information is transmitted from one nerve cell to the next. Synaptically released GABA binds to postsynaptic GABAA receptors, thus causing an influx of negatively charged chloride ions into the postsynaptic neuron. This leads to a hyperpolarization and thus functional inhibition of the postsynaptic cell. Benzodiazepines bind to a site on the GABA_A_ receptor which is different from the GABA binding site, thus increasing the chloride current. Benzodiazepines like diazepam bind to GABA_A_ receptors containing the α subunits α1, α2, α3, or α5, most likely in abgabg combinations.

In a recent study published in the journal *Science* [1], researchers at the University of Zürich and the Swiss Federal Institute (ETH) Zürich have identified the specific subtype of $GABA_A$ receptor that mediates the anxiolytic action of diazepam. In this study, they introduced a histidine to arginine point mutation in the α2 and α3 subunit genes of the mouse [α2(H101R) and α3(H126R) mice]. In a previous study published in *Nature* [2], they had introduced a similar histidine to arginine point mutation in the α1 subunit gene [α1(H101R) mice]. That study revealed that the sedative and the memory-impairing actions of diazepam are mediated by $GABA_A$ receptors containing the α1 subunit. The mediation of the sedative action of diazepam by this receptor subtype was also independently confirmed [3]. However, the anxiolytic and muscle relaxant actions were still present in these α1(H101R) mice [2], indicating that they are not mediated by $GABA_A$ receptors containing a1. The study published in *Science* [1] then identified the α2-containing $GABA_A$ receptors as the mediators of the anxiolytic action of diazepam and demonstrated that α3-containing $GABA_A$ receptors are not involved in this action. Since α2- and α3-containing $GABA_A$ receptors show a distinct distribution pattern in the brain with the α2-containing receptors abundant in limbic areas and the α3-containing receptors being expressed in the brainstem reticular formation, these results also identify the neuronal circuits mediating the anxiolytic action of diazepam.

The past 15 years have seen the molecular cloning and morphological analysis of $GABA_A$ receptor subunits, which led to the realization that multiple subtypes of $GABA_A$

receptors exist. The functional and, in particular, pharmacological significance of this diversity, however, was unknown.

In the study published in *Science* [1], mouse genetics techniques have been used to silence the diazepam response of specific receptor subtypes, namely α2- and α3-containing $GABA_A$ receptors. The introduced histidine to arginine point mutation apparently does not affect the physiological function, but prevents diazepam from binding to these receptor subtypes. Any action of diazepam that is missing in one of the mutated mouse lines indicates that the receptor subtype which is point-mutated in the mutant mice is responsible for mediating this action in the wild type mice. When wild type mice are allowed to explore a lit compartment or an adjacent dark compartment of a box, not surprisingly they will spend most of their time in the dark compartment. The anxiolytic action of diazepam relieved anxiety so that mice spent up to half of the time in the lit compartment. Whereas this anxiolytic action of diazepam is retained in wild type mice and in mice carrying the point mutation in the a1 or α3 subunits, it is missing in mice carrying the point mutation in the α2 subunit, indicating that the α2-containing $GABA_A$ receptors mediate the anxiolytic action of diazepam. In contrast, the sedative action of diazepam is mediated by $GABA_A$ receptors containing α1.

The identification of the $GABA_A$ receptor subtype which is responsible for mediating the anxiolytic action of diazepam identifies the neuronal circuits involved and provides important information for drug development. Currently used benzodiazepines shut down the brain fairly generally producing a variety of undesired effects. Now, with the identification of the therapeutically relevant receptor subtypes, it is possible to design drugs that specifically bind to the $GABA_A$ receptor subtypes that mediate desired drug actions. As a first step in this direction, a compound, L838417, has recently been described in *Nature Neuroscience* [3] which has an agonistic action at α2-, α3-, and α5-containing $GABA_A$ receptors but not at a1-containing $GABA_A$ receptors. This compound has anxiolytic but not sedative actions. The α2-containing $GABA_A$ receptor subtype represents only about 15% of all $GABA_A$ receptors in the brain. We now know that a drug acting only at α2-containing $GABA_A$ receptors most likely would have an anxiolytic but no sedative or anterograde amnesic action. In addition, since such a drug would only affect a small minority of $GABA_A$ receptors, it is conceivable that such a subtype-specific drug might lack other undesired side effects of currently used benzodiazepines.

| | | |
|---|---|---|
| Received: | February | 27, 2001 |
| Accepted: | March | 26, 2001 |
| Published: | May | 1, 2001 |
